# Incidental Diagnosis of Pulmonary Embolism in Asymptomatic Patient Using Endobronchial Ultrasound (EBUS) During Mediastinal Lymphadenopathy Assessment

**DOI:** 10.7759/cureus.13404

**Published:** 2021-02-17

**Authors:** Sherif T Abuserewa, Richard Duff

**Affiliations:** 1 Internal Medicine, Grand Strand Regional Medical Center, Myrtle Beach, USA; 2 Pulmonary and Critical Care Medicine, Grand Strand Medical Center, Myrtle Beach, USA

**Keywords:** pulmonary embolism (pe), ebus, endobronchial ultrasound

## Abstract

The diagnosis of pulmonary embolism (PE) needs clinical manifestations and radiological findings. CT angiography (CTA) of pulmonary vessels is the gold standard of diagnosis of PE. However, endobronchial ultrasound (EBUS) can be a reliable and accurate alternative method of diagnosis in patients who are not candidates for CTA. Invasiveness and high cost are still the major limitations for EBUS, however, they should be considered in the appropriate population in future practice. We present a case of a 62-year-old asymptomatic male diagnosed with PE during EBUS for mediastinal lymph node assessment and biopsy.

## Introduction

Pulmonary embolism is a common thromboembolic disease that usually presents with highly variable clinical manifestations [1-3] and is diagnosed with CT angiography of pulmonary vessels, which is considered the imaging modality of choice [4]. EBUS is usually performed as an adjunct to transbronchial needle aspiration (TBNA) of mediastinal and hilar lymph nodes for the assessment and evaluation of different lymph node pathologies and staging of lung cancer [5-6]. Major pulmonary vessels can be viewed during EBUS and thus pulmonologists should be aware that EBUS can be an alternative modality to diagnose PE in patients who are not candidates for CT pulmonary angiography. We are presenting a case of an asymptomatic male with pulmonary embolism (PE) discovered during EBUS for mediastinal lymph node biopsy.

It is important to mention that this case was presented as an abstract in the 50th Critical Care Congress of the Society of Critical Care Medicine held in January - February 2021.

## Case presentation

A 62-year-old male, former smoker, with a past medical history of large exudative pericardial effusion, epilepsy, and hypothyroidism presented for outpatient EBUS for follow-up of positron emission tomography (PET)-avid mediastinal and hilar lymphadenopathy that were found during the workup of his pericardial effusion. The patient was physically active walking daily without chest pain or dyspnea. He reported having a non-productive cough. On examination, the patient was alert, sitting comfortably, without lower extremities edema, and his vitals were stable. Chest auscultation revealed loud S2 in the pulmonary area. All of his blood tests, including troponin I and pro-B-type natriuretic peptide (pro-BNP) were completely normal. His electrocardiogram (EKG) showed normal sinus rhythm.

**Figure 1:**
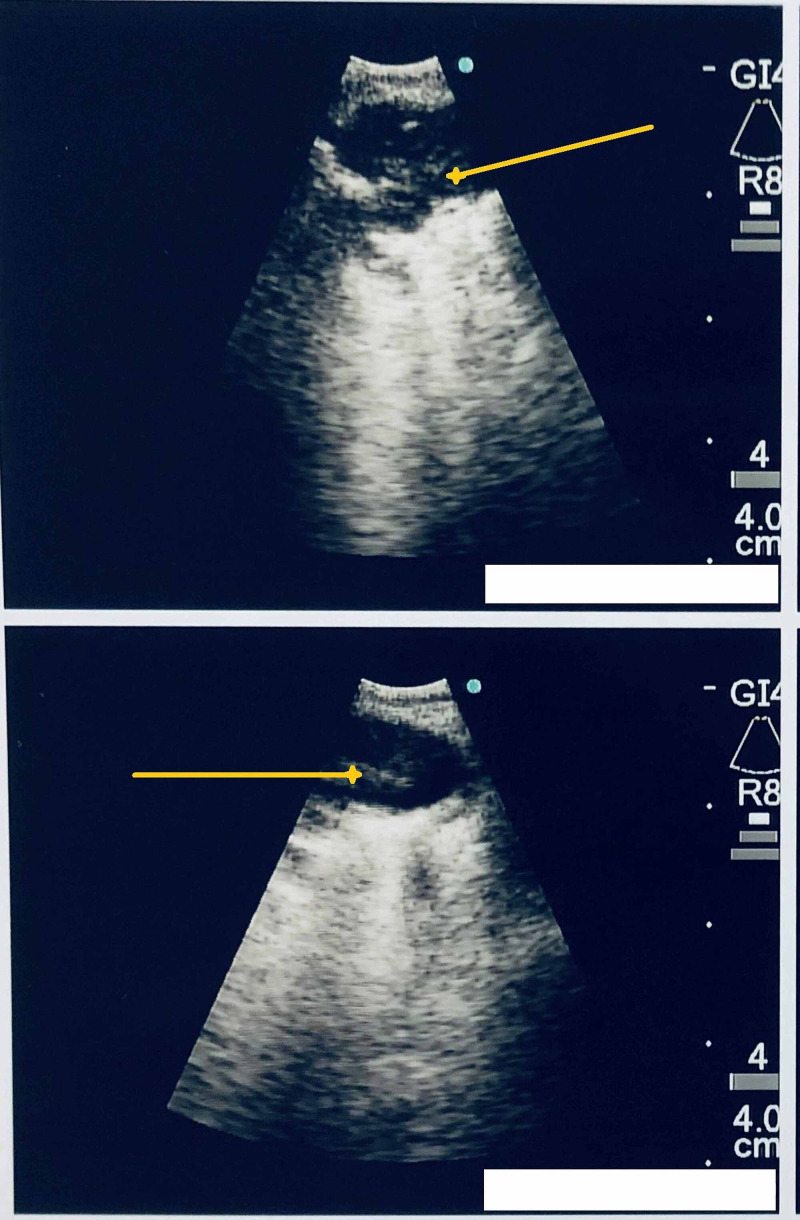
EBUS showing floating thrombus (pointed by yellow arrows) in the pulmonary artery lumen EBUS: endobronchial ultrasound

**Figure 2:**
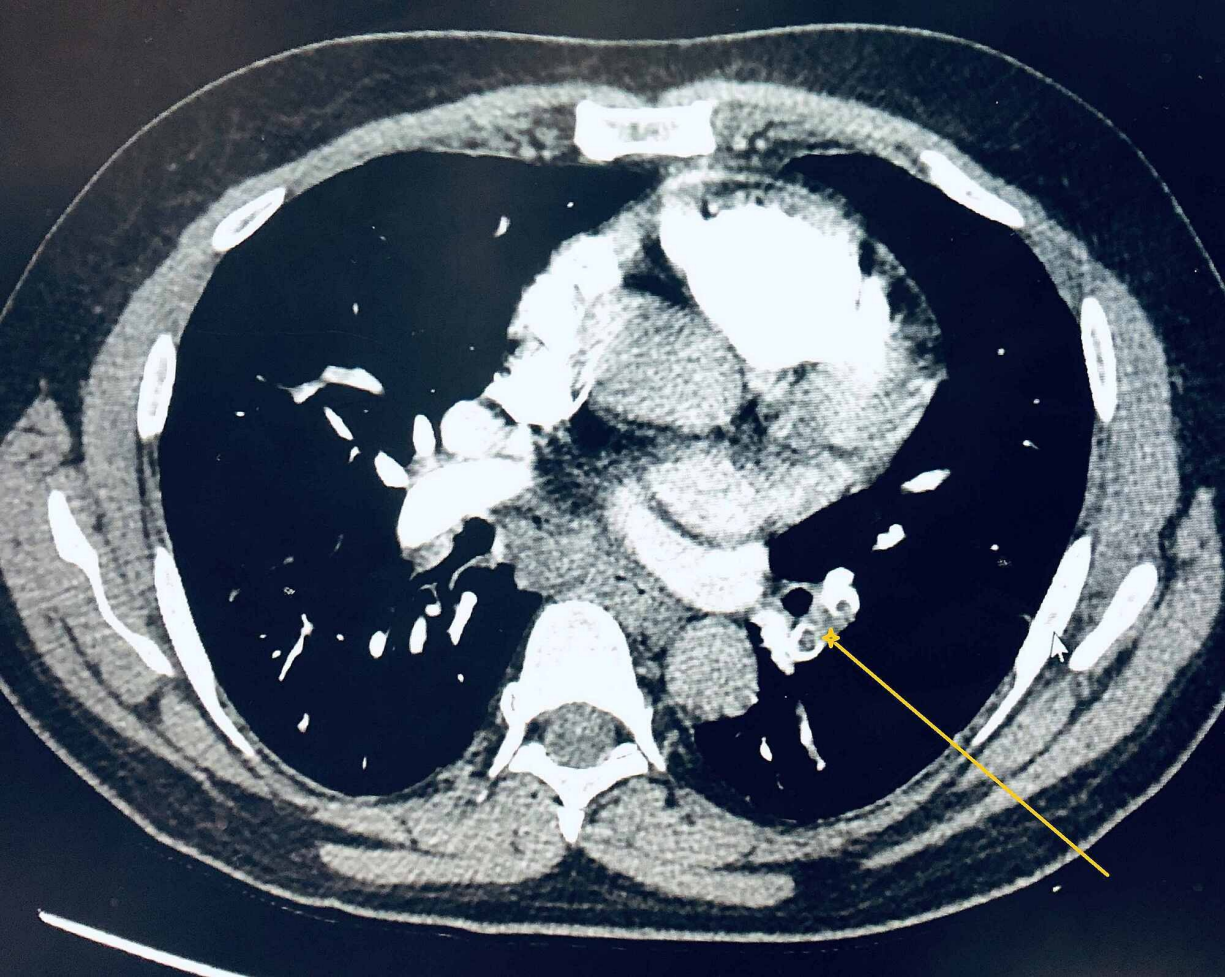
CTA chest axial view revealing acute appearing pulmonary emboli (pointed by yellow arrow) identified throughout multiple segmental branches of the left lower lobe pulmonary artery CTA: computed tomography angiography

**Figure 3:**
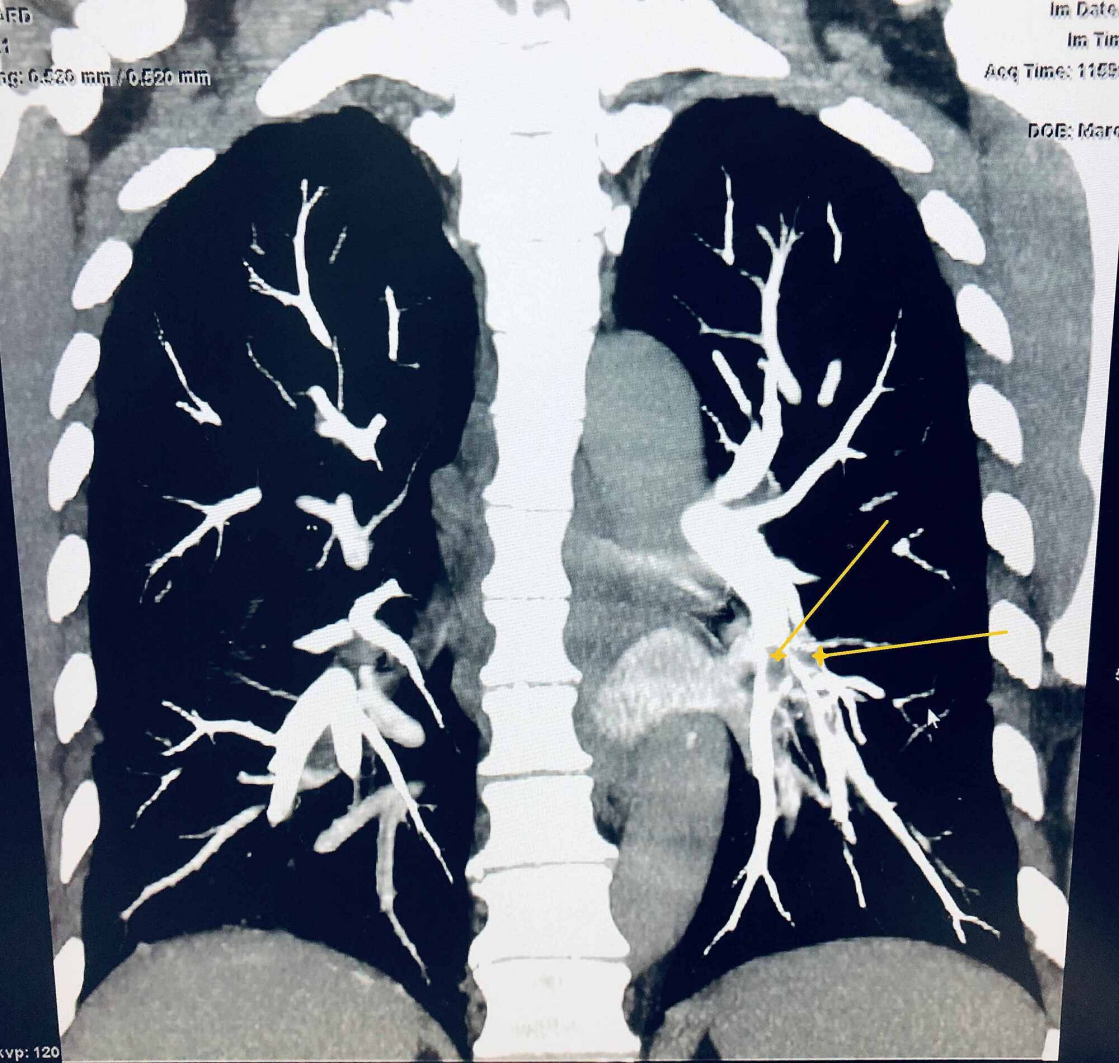
CTA chest coronal view revealing acute appearing pulmonary emboli (pointed by yellow arrows) identified throughout multiple segmental branches of the left lower lobe pulmonary artery. CTA: computed tomography angiography

During EBUS, an abnormal floating object was visualized in a vascular structure of left pulmonary vessels as shown in Figure 1. After the procedure was completed with several biopsies taken of stations 7, 10, and 11, the procedure was aborted and the patient went for emergent computed tomography angiography (CTA) revealing acute appearing pulmonary emboli identified throughout multiple segmental branches of the left lower lobe pulmonary artery as shown in Figures 2-3. An echocardiogram was done and showed ejection fraction (EF) 70% or greater with no pericardial effusion or signs of right ventricular (RV) strain. The patient was discharged on apixaban.

## Discussion

A pulmonary embolism is a life-threatening pulmonary vascular thromboembolic disease that affects around 900,000 people (1 to 2 per 1,000) each year in the United States, with no sex predilection, and it usually results in between 60,000 and 100,000 deaths per year according to the Centers for Disease Control and Prevention (CDC) [7-8]. It has multiple etiologies, which are usually fulfilling the criteria of Virchow’s triad, i.e., hypercoagulability, endothelial injury, and bloodstream stagnation. It presents with highly variable clinical manifestations, including dyspnea, tachypnea, pleuritic chest pain, cough, and hemoptysis, and in more severe cases, may present with cyanosis and collapse [1-3,9-10].

A pulmonary embolism is a critical condition that requires rapid diagnosis and treatment for favorable outcomes. CT pulmonary angiography is considered the diagnostic imaging modality of choice due to its accuracy, non-invasive nature, its availability, and its ability to identify other lung pathologies [4,11]. Although ventilation/perfusion scan has the same accuracy as CT pulmonary angiography and can be used in pregnant women, patients with renal failure, and patients allergic to the intravenous (IV) contrast, it is less utilized due to the advanced technology needed for it to be processed [10,12].

EBUS is usually performed as an adjunct to transbronchial needle aspiration (TBNA) of mediastinal and hilar lymph nodes for the assessment and evaluation of different lymph node pathologies and the staging of lung cancer. The anatomical proximity of the bronchial airways and the pulmonary vasculature allows the EBUS theoretically to evaluate the pulmonary arteries for any abnormalities or pulmonary embolism [13]. Several studies have been done to assess the accuracy of EBUS to diagnose PE in comparison to CT pulmonary angiography. In one pilot study on intensive care unit (ICU) patients who were already diagnosed with PE by CT pulmonary angiography, EBUS was able to identify 96% of thrombi identified by CTA [14]. In another study, EBUS was more accurate to identify PE, which was not detected by CTA [15]. In some situations, CT pulmonary angiography are difficult to be done as the patient may be too unstable for transport to the CT scanner or not a candidate to receive IV contrast due to allergy, pregnancy, or renal insufficiency, and here EBUS may be used to diagnose PE. EBUS has also the ability to differentiate between PE and pulmonary sarcoma [16] or hilar abnormality draping over the pulmonary artery [17], which are all difficult to be distinguished on CTA. Despite the previously mentioned potential advantages of EBUS in the ICU population, it will not be practical to be used in the general population for the diagnosis of PE because of invasiveness and high cost, however, it should be considered in the appropriate population in the future practice.

## Conclusions

Major pulmonary vessels can be viewed during EBUS, and thus pulmonologists should be aware that EBUS can be a good alternative modality to diagnose PE with high sensitivity and specificity in patients who are not candidates for CT pulmonary angiography. More studies are needed for the further evaluation of the diagnostic value of EBUS for PE.
